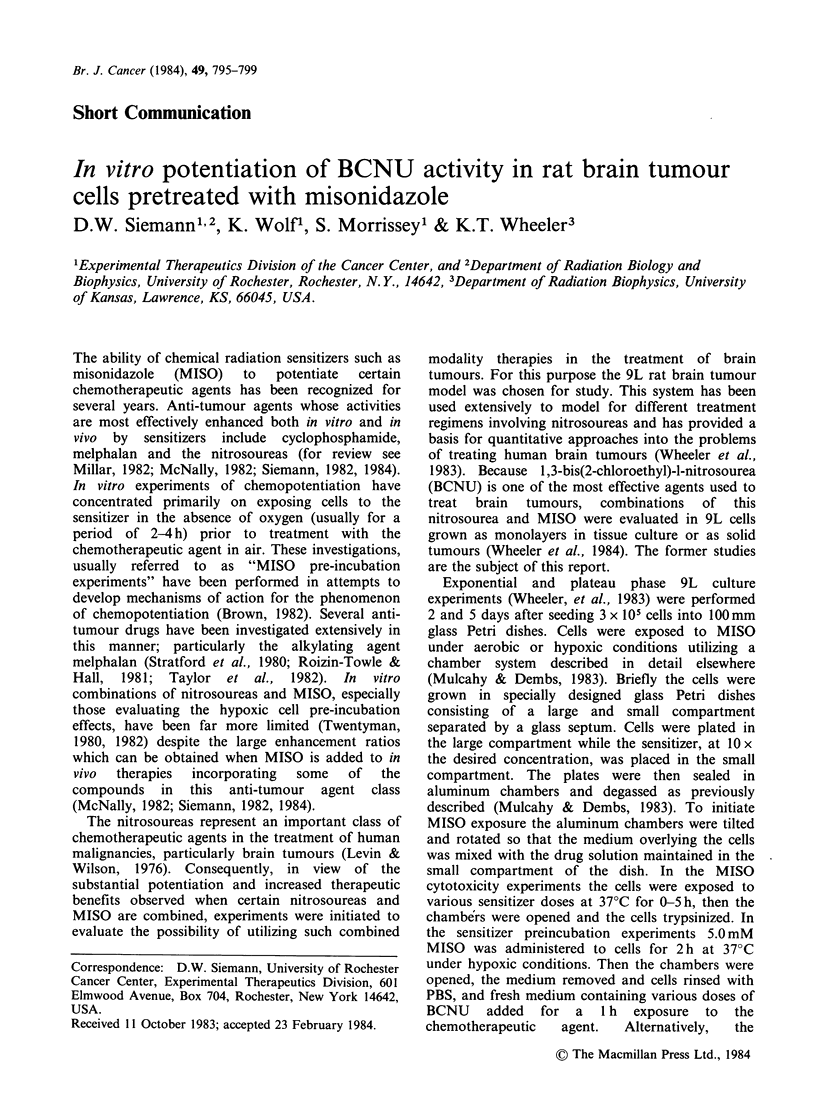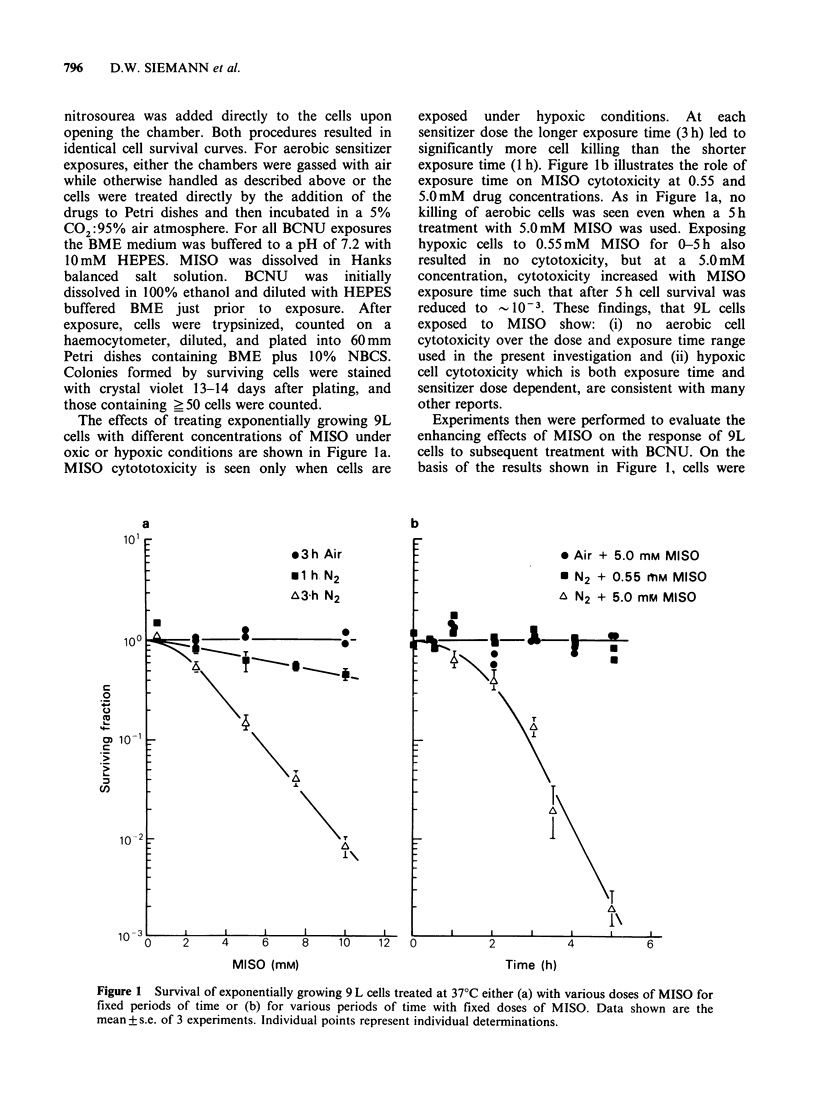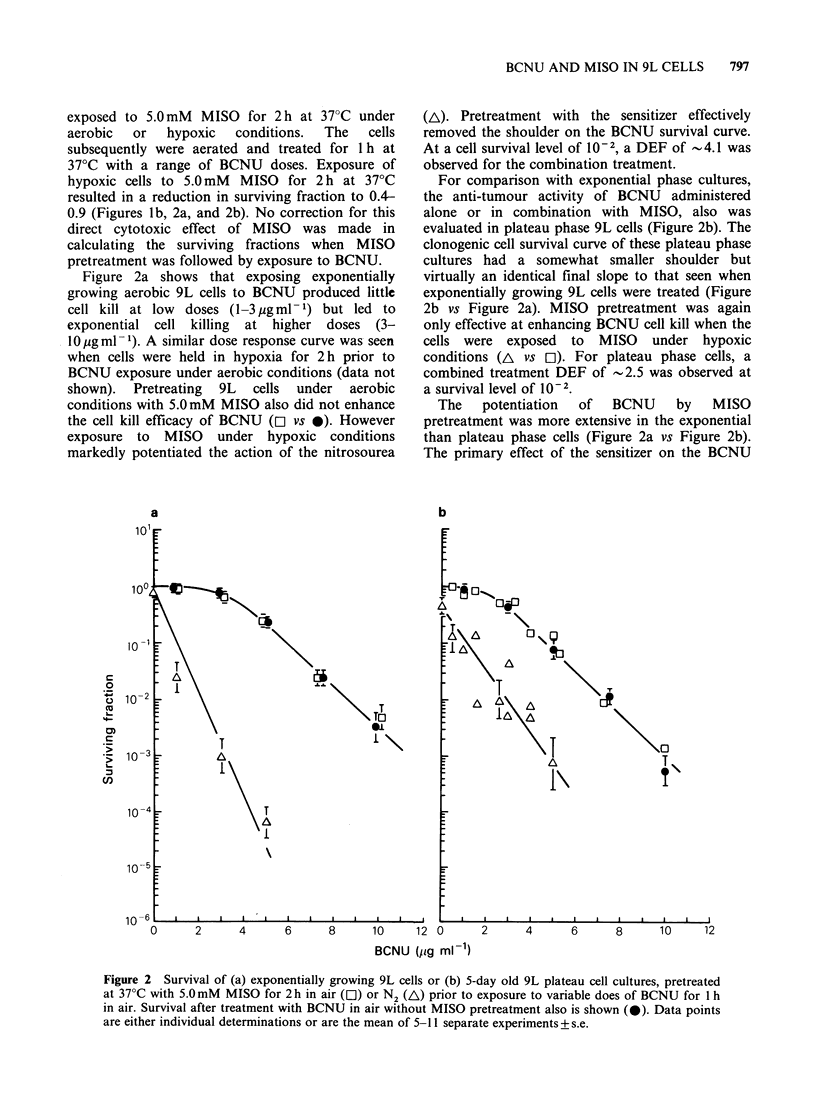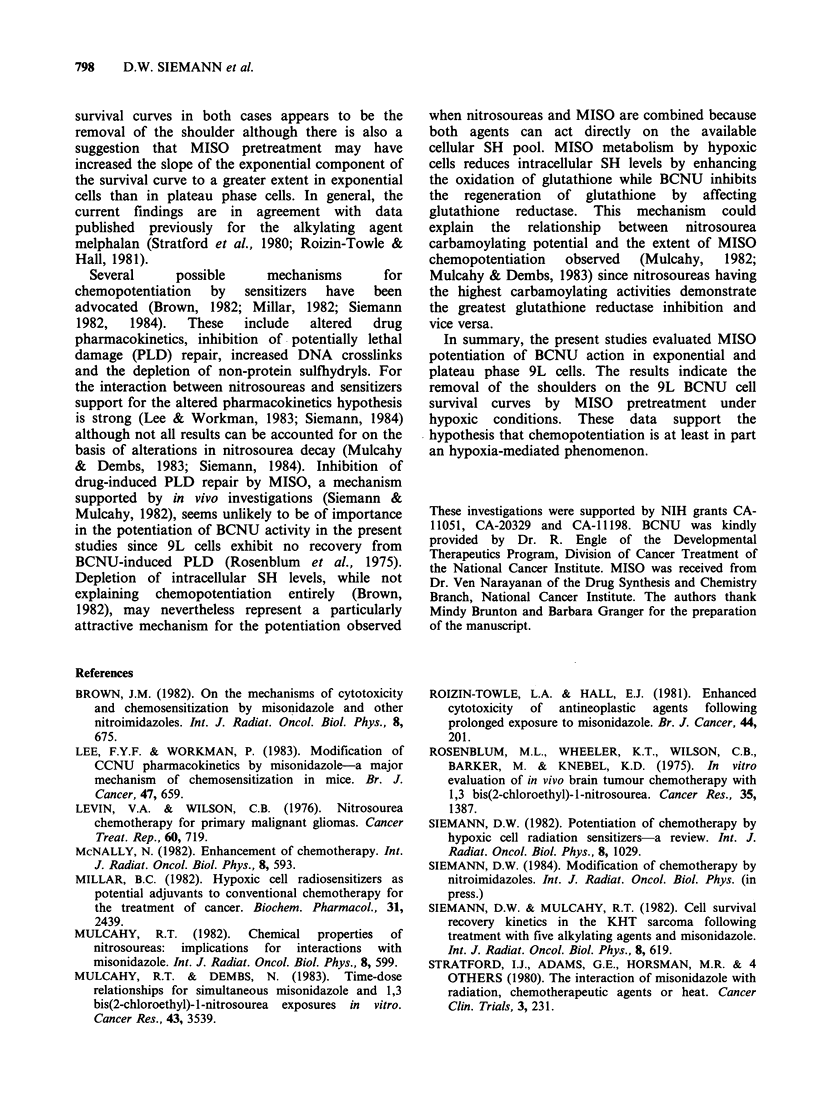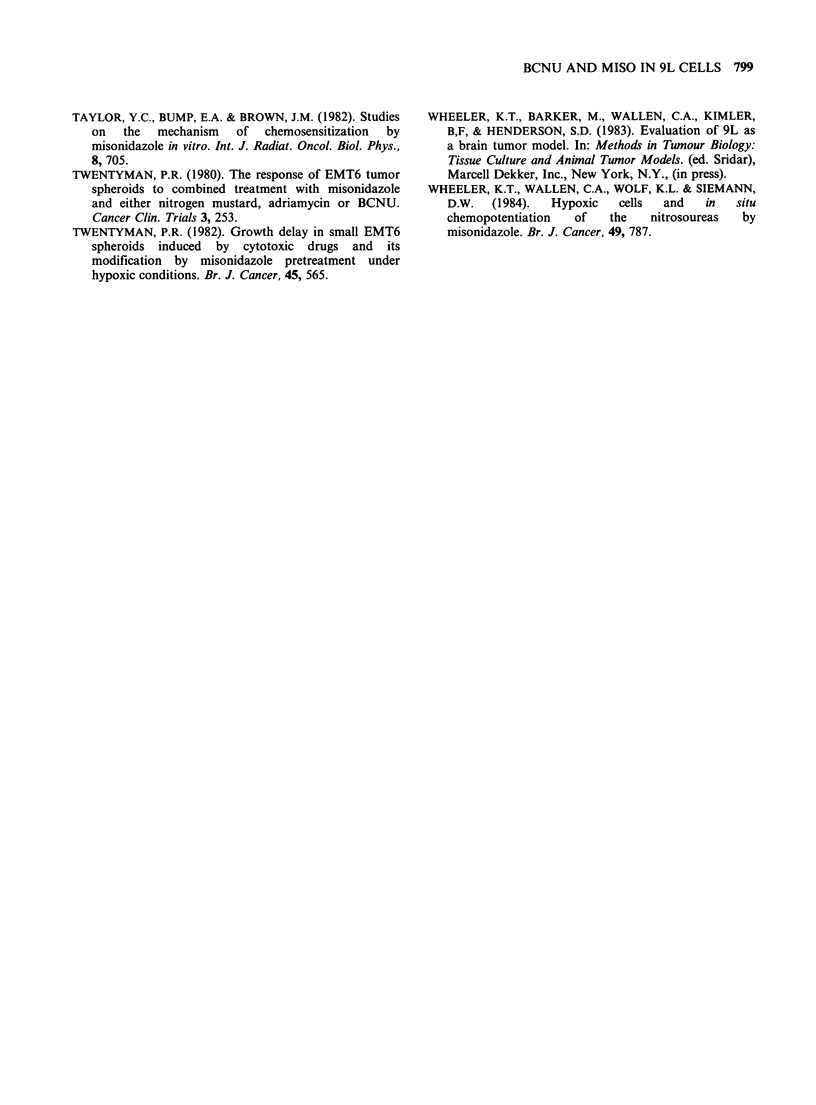# In vitro potentiation of BCNU activity in rat brain tumour cells pretreated with misonidazole.

**DOI:** 10.1038/bjc.1984.123

**Published:** 1984-06

**Authors:** D. W. Siemann, K. Wolf, S. Morrissey, K. T. Wheeler


Br. J. Cancer (1984), **49**, 795–799

### Short Communication

### *In vitro* potentiation of BCNU activity in rat brain tumour cells pretreated with misonidazole

D.W. Siemann[1,2], K. Wolf[1], S. Morrissey[1] & K.T. Wheeler[3]

[1]*Experimental Therapeutics Division of the Cancer Center, and* [2]*Department of Radiation Biology and Biophysics, University of Rochester, Rochester, N.Y., 14642,* [3]*Department of Radiation Biophysics, University of Kansas, Lawrence, KS, 66045, USA.*

The ability of chemical radiation sensitizers such as misonidazole (MISO) to potentiate certain chemotherapeutic agents has been recognized for several years. Anti-tumour agents whose activities are most effectively enhanced both *in vitro* and *in vivo* by sensitizers include cyclophosphamide, melphalan and the nitrosoureas (for review see Millar, 1982; McNally, 1982; Siemann, 1982, 1984). *In vitro* experiments of chemopotentiation have concentrated primarily on exposing cells to the sensitizer in the absence of oxygen (usually for a period of 2–4 h) prior to treatment with the chemotherapeutic agent in air. These investigations, usually referred to as "MISO pre-incubation experiments" have been performed in attempts to develop mechanisms of action for the phenomenon of chemopotentiation (Brown, 1982). Several anti-tumour drugs have been investigated extensively in this manner; particularly the alkylating agent melphalan (Stratford *et al.*, 1980; Roizin-Towle & Hall, 1981; Taylor *et al.*, 1982). *In vitro* combinations of nitrosoureas and MISO, especially those evaluating the hypoxic cell pre-incubation effects, have been far more limited (Twentyman, 1980, 1982) despite the large enhancement ratios which can be obtained when MISO is added to *in vivo* therapies incorporating some of the compounds in this anti-tumour agent class (McNally, 1982; Siemann, 1982, 1984).

The nitrosoureas represent an important class of chemotherapeutic agents in the treatment of human malignancies, particularly brain tumours (Levin & Wilson, 1976). Consequently, in view of the substantial potentiation and increased therapeutic benefits observed when certain nitrosoureas and MISO are combined, experiments were initiated to evaluate the possibility of utilizing such combined modality therapies in the treatment of brain tumours. For this purpose the 9L rat brain tumour model was chosen for study. This system has been used extensively to model for different treatment regimens involving nitrosoureas and has provided a basis for quantitative approaches into the problems of treating human brain tumours (Wheeler *et al.*, 1983). Because 1,3-bis(2-chloroethyl)-l-nitrosourea (BCNU) is one of the most effective agents used to treat brain tumours, combinations of this nitrosourea and MISO were evaluated in 9L cells grown as monolayers in tissue culture or as solid tumours (Wheeler *et al.*, 1984). The former studies are the subject of this report.

Exponential and plateau phase 9L culture experiments (Wheeler, *et al.*, 1983) were performed 2 and 5 days after seeding $3 \times 10^5$ cells into 100 mm glass Petri dishes. Cells were exposed to MISO under aerobic or hypoxic conditions utilizing a chamber system described in detail elsewhere (Mulcahy & Dembs, 1983). Briefly the cells were grown in specially designed glass Petri dishes consisting of a large and small compartment separated by a glass septum. Cells were plated in the large compartment while the sensitizer, at $10 \times$ the desired concentration, was placed in the small compartment. The plates were then sealed in aluminum chambers and degassed as previously described (Mulcahy & Dembs, 1983). To initiate MISO exposure the aluminum chambers were tilted and rotated so that the medium overlying the cells was mixed with the drug solution maintained in the small compartment of the dish. In the MISO cytotoxicity experiments the cells were exposed to various sensitizer doses at 37°C for 0–5 h, then the chambers were opened and the cells trypsinized. In the sensitizer preincubation experiments 5.0 mM MISO was administered to cells for 2 h at 37°C under hypoxic conditions. Then the chambers were opened, the medium removed and cells rinsed with PBS, and fresh medium containing various doses of BCNU added for a 1 h exposure to the chemotherapeutic agent. Alternatively, the

Correspondence: D.W. Siemann, University of Rochester Cancer Center, Experimental Therapeutics Division, 601 Elmwood Avenue, Box 704, Rochester, New York 14642, USA.
Received 11 October 1983; accepted 23 February 1984.

© The Macmillan Press Ltd., 1984

nitrosourea was added directly to the cells upon opening the chamber. Both procedures resulted in identical cell survival curves. For aerobic sensitizer exposures, either the chambers were gassed with air while otherwise handled as described above or the cells were treated directly by the addition of the drugs to Petri dishes and then incubated in a 5% $CO_2$:95% air atmosphere. For all BCNU exposures the BME medium was buffered to a pH of 7.2 with 10 mM HEPES. MISO was dissolved in Hanks balanced salt solution. BCNU was initially dissolved in 100% ethanol and diluted with HEPES buffered BME just prior to exposure. After exposure, cells were trypsinized, counted on a haemocytometer, diluted, and plated into 60 mm Petri dishes containing BME plus 10% NBCS. Colonies formed by surviving cells were stained with crystal violet 13–14 days after plating, and those containing ≧50 cells were counted.

The effects of treating exponentially growing 9L cells with different concentrations of MISO under oxic or hypoxic conditions are shown in Figure 1a. MISO cytototoxicity is seen only when cells are exposed under hypoxic conditions. At each sensitizer dose the longer exposure time (3 h) led to significantly more cell killing than the shorter exposure time (1 h). Figure 1b illustrates the role of exposure time on MISO cytotoxicity at 0.55 and 5.0 mM drug concentrations. As in Figure 1a, no killing of aerobic cells was seen even when a 5 h treatment with 5.0 mM MISO was used. Exposing hypoxic cells to 0.55 mM MISO for 0–5 h also resulted in no cytotoxicity, but at a 5.0 mM concentration, cytotoxicity increased with MISO exposure time such that after 5 h cell survival was reduced to $\sim 10^{-3}$. These findings, that 9L cells exposed to MISO show: (i) no aerobic cell cytotoxicity over the dose and exposure time range used in the present investigation and (ii) hypoxic cell cytotoxicity which is both exposure time and sensitizer dose dependent, are consistent with many other reports.

Experiments then were performed to evaluate the enhancing effects of MISO on the response of 9L cells to subsequent treatment with BCNU. On the basis of the results shown in Figure 1, cells were

**Figure 1** Survival of exponentially growing 9 L cells treated at 37°C either (a) with various doses of MISO for fixed periods of time or (b) for various periods of time with fixed doses of MISO. Data shown are the mean ± s.e. of 3 experiments. Individual points represent individual determinations.

exposed to 5.0 mM MISO for 2 h at 37°C under aerobic or hypoxic conditions. The cells subsequently were aerated and treated for 1 h at 37°C with a range of BCNU doses. Exposure of hypoxic cells to 5.0 mM MISO for 2 h at 37°C resulted in a reduction in surviving fraction to 0.4–0.9 (Figures 1b, 2a, and 2b). No correction for this direct cytotoxic effect of MISO was made in calculating the surviving fractions when MISO pretreatment was followed by exposure to BCNU.

Figure 2a shows that exposing exponentially growing aerobic 9L cells to BCNU produced little cell kill at low doses (1–3 $\mu g\,ml^{-1}$) but led to exponential cell killing at higher doses (3–10 $\mu g\,ml^{-1}$). A similar dose response curve was seen when cells were held in hypoxia for 2 h prior to BCNU exposure under aerobic conditions (data not shown). Pretreating 9L cells under aerobic conditions with 5.0 mM MISO also did not enhance the cell kill efficacy of BCNU (□ *vs* ●). However exposure to MISO under hypoxic conditions markedly potentiated the action of the nitrosourea (△). Pretreatment with the sensitizer effectively removed the shoulder on the BCNU survival curve. At a cell survival level of $10^{-2}$, a DEF of ~4.1 was observed for the combination treatment.

For comparison with exponential phase cultures, the anti-tumour activity of BCNU administered alone or in combination with MISO, also was evaluated in plateau phase 9L cells (Figure 2b). The clonogenic cell survival curve of these plateau phase cultures had a somewhat smaller shoulder but virtually an identical final slope to that seen when exponentially growing 9L cells were treated (Figure 2b *vs* Figure 2a). MISO pretreatment was again only effective at enhancing BCNU cell kill when the cells were exposed to MISO under hypoxic conditions (△ *vs* □). For plateau phase cells, a combined treatment DEF of ~2.5 was observed at a survival level of $10^{-2}$.

The potentiation of BCNU by MISO pretreatment was more extensive in the exponential than plateau phase cells (Figure 2a *vs* Figure 2b). The primary effect of the sensitizer on the BCNU

**Figure 2** Survival of (a) exponentially growing 9L cells or (b) 5-day old 9L plateau cell cultures, pretreated at 37°C with 5.0 mM MISO for 2 h in air (□) or $N_2$ (△) prior to exposure to variable does of BCNU for 1 h in air. Survival after treatment with BCNU in air without MISO pretreatment also is shown (●). Data points are either individual determinations or are the mean of 5–11 separate experiments ± s.e.

survival curves in both cases appears to be the removal of the shoulder although there is also a suggestion that MISO pretreatment may have increased the slope of the exponential component of the survival curve to a greater extent in exponential cells than in plateau phase cells. In general, the current findings are in agreement with data published previously for the alkylating agent melphalan (Stratford *et al.*, 1980; Roizin-Towle & Hall, 1981).

Several possible mechanisms for chemopotentiation by sensitizers have been advocated (Brown, 1982; Millar, 1982; Siemann 1982, 1984). These include altered drug pharmacokinetics, inhibition of potentially lethal damage (PLD) repair, increased DNA crosslinks and the depletion of non-protein sulfhydryls. For the interaction between nitrosoureas and sensitizers support for the altered pharmacokinetics hypothesis is strong (Lee & Workman, 1983; Siemann, 1984) although not all results can be accounted for on the basis of alterations in nitrosourea decay (Mulcahy & Dembs, 1983; Siemann, 1984). Inhibition of drug-induced PLD repair by MISO, a mechanism supported by *in vivo* investigations (Siemann & Mulcahy, 1982), seems unlikely to be of importance in the potentiation of BCNU activity in the present studies since 9L cells exhibit no recovery from BCNU-induced PLD (Rosenblum *et al.*, 1975). Depletion of intracellular SH levels, while not explaining chemopotentiation entirely (Brown, 1982), may nevertheless represent a particularly attractive mechanism for the potentiation observed when nitrosoureas and MISO are combined because both agents can act directly on the available cellular SH pool. MISO metabolism by hypoxic cells reduces intracellular SH levels by enhancing the oxidation of glutathione while BCNU inhibits the regeneration of glutathione by affecting glutathione reductase. This mechanism could explain the relationship between nitrosourea carbamoylating potential and the extent of MISO chemopotentiation observed (Mulcahy, 1982; Mulcahy & Dembs, 1983) since nitrosoureas having the highest carbamoylating activities demonstrate the greatest glutathione reductase inhibition and vice versa.

In summary, the present studies evaluated MISO potentiation of BCNU action in exponential and plateau phase 9L cells. The results indicate the removal of the shoulders on the 9L BCNU cell survival curves by MISO pretreatment under hypoxic conditions. These data support the hypothesis that chemopotentiation is at least in part an hypoxia-mediated phenomenon.

These investigations were supported by NIH grants CA-11051, CA-20329 and CA-11198. BCNU was kindly provided by Dr. R. Engle of the Developmental Therapeutics Program, Division of Cancer Treatment of the National Cancer Institute. MISO was received from Dr. Ven Narayanan of the Drug Synthesis and Chemistry Branch, National Cancer Institute. The authors thank Mindy Brunton and Barbara Granger for the preparation of the manuscript.

### References

BROWN, J.M. (1982). On the mechanisms of cytotoxicity and chemosensitization by misonidazole and other nitroimidazoles. *Int. J. Radiat. Oncol. Biol. Phys.*, **8**, 675.

LEE, F.Y.F. & WORKMAN, P. (1983). Modification of CCNU pharmacokinetics by misonidazole—a major mechanism of chemosensitization in mice. *Br. J. Cancer*, **47**, 659.

LEVIN, V.A. & WILSON, C.B. (1976). Nitrosourea chemotherapy for primary malignant gliomas. *Cancer Treat. Rep.*, **60**, 719.

McNALLY, N. (1982). Enhancement of chemotherapy. *Int. J. Radiat. Oncol. Biol. Phys.*, **8**, 593.

MILLAR, B.C. (1982). Hypoxic cell radiosensitizers as potential adjuvants to conventional chemotherapy for the treatment of cancer. *Biochem. Pharmacol.*, **31**, 2439.

MULCAHY, R.T. (1982). Chemical properties of nitrosoureas: implications for interactions with misonidazole. *Int. J. Radiat. Oncol. Biol. Phys.*, **8**, 599.

MULCAHY, R.T. & DEMBS, N. (1983). Time-dose relationships for simultaneous misonidazole and 1,3 bis(2-chloroethyl)-1-nitrosourea exposures *in vitro*. *Cancer Res.*, **43**, 3539.

ROIZIN-TOWLE, L.A. & HALL, E.J. (1981). Enhanced cytotoxicity of antineoplastic agents following prolonged exposure to misonidazole. *Br. J. Cancer*, **44**, 201.

ROSENBLUM, M.L., WHEELER, K.T., WILSON, C.B., BARKER, M. & KNEBEL, K.D. (1975). *In vitro* evaluation of *in vivo* brain tumour chemotherapy with 1,3 bis(2-chloroethyl)-1-nitrosourea. *Cancer Res.*, **35**, 1387.

SIEMANN, D.W. (1982). Potentiation of chemotherapy by hypoxic cell radiation sensitizers—a review. *Int. J. Radiat. Oncol. Biol. Phys.*, **8**, 1029.

SIEMANN, D.W. (1984). Modification of chemotherapy by nitroimidazoles. *Int. J. Radiat. Oncol. Biol. Phys.* (in press.)

SIEMANN, D.W. & MULCAHY, R.T. (1982). Cell survival recovery kinetics in the KHT sarcoma following treatment with five alkylating agents and misonidazole. *Int. J. Radiat. Oncol. Biol. Phys.*, **8**, 619.

STRATFORD, I.J., ADAMS, G.E., HORSMAN, M.R. & 4 OTHERS (1980). The interaction of misonidazole with radiation, chemotherapeutic agents or heat. *Cancer Clin. Trials*, **3**, 231.

TAYLOR, Y.C., BUMP, E.A. & BROWN, J.M. (1982). Studies on the mechanism of chemosensitization by misonidazole *in vitro*. *Int. J. Radiat. Oncol. Biol. Phys.*, **8**, 705.

TWENTYMAN, P.R. (1980). The response of EMT6 tumor spheroids to combined treatment with misonidazole and either nitrogen mustard, adriamycin or BCNU. *Cancer Clin. Trials* **3**, 253.

TWENTYMAN, P.R. (1982). Growth delay in small EMT6 spheroids induced by cytotoxic drugs and its modification by misonidazole pretreatment under hypoxic conditions. *Br. J. Cancer*, **45**, 565.

WHEELER, K.T., BARKER, M., WALLEN, C.A., KIMLER, B,F, & HENDERSON, S.D. (1983). Evaluation of 9L as a brain tumor model. In: *Methods in Tumour Biology: Tissue Culture and Animal Tumor Models*. (ed. Sridar), Marcell Dekker, Inc., New York, N.Y., (in press).

WHEELER, K.T., WALLEN, C.A., WOLF, K.L. & SIEMANN, D.W. (1984). Hypoxic cells and *in situ* chemopotentiation of the nitrosoureas by misonidazole. *Br. J. Cancer*, **49**, 787.